# Hyaluronan Regulates Bone Morphogenetic Protein-7-dependent Prevention and Reversal of Myofibroblast Phenotype*

**DOI:** 10.1074/jbc.M114.625939

**Published:** 2015-02-25

**Authors:** Adam C. Midgley, Lucy Duggal, Robert Jenkins, Vincent Hascall, Robert Steadman, Aled O. Phillips, Soma Meran

**Affiliations:** From the ‡ Institute of Nephrology, Institute of Molecular and Experimental Medicine, School of Medicine, Cardiff University, Cardiff CF14 4XN, Wales, United Kingdom and; the § Lerner Research Institute, Department of Biomedical Engineering, Cleveland Clinic, Cleveland, Ohio 44195

**Keywords:** Bone Morphogenetic Protein (BMP), CD44, Hyaluronan, Hyaluronidase, Myofibroblast

## Abstract

Hyaluronan (HA) promotes transforming growth factor (TGF)-β1-driven myofibroblast phenotype. However, HA can also have disease-limiting activity. Bone morphogenetic protein-7 (BMP7) is an antifibrotic cytokine that antagonizes TGF-β1, and isolated studies have demonstrated that HA can both mediate and modulate BMP7 responses. In this study, we investigated whether BMP7 can modulate HA in a manner that leads to prevention/reversal of TGF-β1-driven myofibroblast differentiation in human lung fibroblasts. Results demonstrated that BMP7 prevented and reversed TGF-β1-driven myofibroblast differentiation through a novel mechanism. BMP7 promoted the dissolution and internalization of cell-surface HA into cytoplasmic endosomes. Endosomal HA co-localized with the HA-degrading enzymes, hyaluronidase-1 and hyaluronidase-2 (Hyal2). Moreover, BMP7 showed differential regulation of CD44 standard and variant isoform expression, when compared with TGF-β1. In particular, BMP7 increased membrane expression of CD44v7/8. Inhibiting CD44v7/8 as well as blocking Hyal2 and the Na^+^/H^+^ exchanger-1 at the cell-surface prevented BMP7-driven HA internalization and BMP7-mediated prevention/reversal of myofibroblast phenotype. In summary, a novel mechanism of TGF-β1 antagonism by BMP7 is shown and identifies alteration in HA as critical in mediating BMP7 responses. In addition, we identify Hyal2 and CD44v7/8 as new potential targets for manipulation in prevention and reversal of fibrotic pathology.

## Teaser

**Background:** Bone morphogenetic protein-7 (BMP7) can prevent/reverse fibrosis, but mechanisms are unclear.

**Results:** BMP7 prevents/reverses myofibroblast formation through cell-surface hyaluronan internalization into catalytic endosomes. Hyaluronidase-2 and variant CD44v7/8 mediate this process.

**Conclusion:** Alterations in hyaluronan are critical in prevention/reversal of myofibroblast differentiation.

**Significance:** A novel mechanism of BMP7 action is described and identifies CD44v7/8 and hyaluronidase-2 as potential targets in preventing fibrotic pathology.

## Introduction

Bone morphogenetic protein-7 (BMP7) is a member of the transforming growth factor-β (TGF-β) superfamily that demonstrates reduced expression in progressive renal diseases. Studies in animal models of renal disease, including ischemic reperfusion injury, unilateral urethral obstruction, nephrotoxin-induced glomerulonephritis, and streptozotocin-induced diabetic nephropathy, have shown that BMP7 can both prevent and reverse renal fibrosis (1–4). Studies in animal models of pulmonary and liver fibrosis have demonstrated similar benefits (5–7). However, BMP7 has also been implicated in progression of bone metastasis in osteotropic cancers and in promoting ectopic bone formation, and thus it is not currently viable as a clinical therapy (8). Dissecting the mechanisms by which BMP7 prevents and reverses fibrosis may provide more suitable therapeutic targets. There are data indicating that the actions of BMP7 are, in part, related to antagonism of the biological effects of TGF-β1, a pro-fibrotic mediator (9–11). BMP7 has been shown to attenuate TGF-β1-dependent Smad3 signaling (10). However, other studies have shown that Smad-independent, MAPK-signaling events are central to TGF-β1-driven renal fibrosis (11, 12). Hence, the anti-fibrotic mechanisms of BMP7 are not yet fully elucidated.

Hyaluronan (HA)^2^ is a connective tissue glycosaminoglycan, which *in vivo* is present as a high molecular mass component of extracellular matrices. It is synthesized by three mammalian HA synthase isoenzymes (HAS1, HAS2, and HAS3), and its breakdown is mediated by the hyaluronidase (Hyal) group of enzymes. HA regulates cellular processes through interaction with cell-surface receptors (principally CD44) and has been implicated in numerous biological processes, including embryonic development, wound healing, chronic inflammation, and tumor progression (13–16). Therefore, HA has an important role in maintaining tissue homeostasis.

Alterations in HA generation and turnover have also been associated with promotion of disease states. Increased expression of HA has been demonstrated in numerous fibrotic conditions associated with organ dysfunction, including IgA nephropathy, diabetic nephropathy, pulmonary fibrosis, and cirrhotic liver disease (17–24). We have previously shown that changes in HA synthesis and macromolecular organization are key in directing TGF-β1-driven differentiation of fibroblasts to myofibroblasts, the principal effector cells in fibrosis (12, 25–27). TGF-β1 stimulation directs HAS2-driven assembly of HA into pericellular coats, which promotes membrane redistribution of CD44. This results in its physical co-localization with the epidermal growth factor receptor (EGFR) and subsequent selective activation of the MAPK/ERK signaling pathway necessary for myofibroblast formation (12, 27). Numerous reports have indicated that HA assembly and its conformation within the pericellular matrix are crucial in determining its subsequent biological actions. For example, interleukin-1β (IL-1β)-mediated formation of HA pericellular protrusions promotes fibroblast immune activation through fibroblast-monocyte interactions, whereas pericellular HA cable-like structures are shown to be anti-inflammatory and prevent monocyte-dependent inflammatory cytokine production (28, 29). Thus, it is clear that under specific biological contexts HA may also have the ability to function in the prevention of disease.

There is some evidence that HA can both mediate and modulate BMP7 responses. In chondrocytes, BMP7-dependent Smad1 signaling was mediated through CD44-Smad1 interactions (30). Other studies demonstrated that pericellular HA augments BMP7 Smad signaling (31). In renal epithelial cells, BMP7 has also been shown to induce the formation of HA cables (32). However, whether HA plays a specific role in BMP7-mediated antagonism of TGF-β1 actions is not known. In this work, we demonstrate a novel mechanism in which BMP7 reverses TGF-β1 induction of myofibroblast differentiation in human lung fibroblast cultures by internalizing pericellular HA into catalytic endosomes within the cytoplasm and preventing TGF-β1-driven pericellular HA accumulation. The CD44 variant isoform, CD44v7/8, and membrane-bound Hyal2 are both critical for this process of HA internalization and thus potentially present new targets for study and intervention in fibrosis.

## EXPERIMENTAL PROCEDURES

### 

#### 

##### Materials

All reagents were purchased from Sigma (Poole, UK) or Life Technologies and Invitrogen (Paisley, UK) unless otherwise stated. Reverse transcription reagents, siRNA transfection reagents, and quantitative PCR (QPCR) primers and reagents were purchased from Invitrogen and Applied Biosystems (Cheshire, UK). Other reagents used were recombinant human BMP7 (Merck Millipore, Darmstadt, Germany), recombinant human TGF-β1 (R&D Systems, Abingdon, UK), and the selective NHE1 inhibitor, 5-(*N*-ethyl-*N*-isopropyl) amiloride (EIPA; Sigma).

##### Cell Culture

Human lung fibroblasts (AG02262) were purchased from Coriell Cell Repositories (Coriell Institute for Medical Research, Camden, NJ). The cells were cultured in Dulbecco's modified Eagle's medium (DMEM) and F12 containing 5 mm glucose, 2 mmol/liter l-glutamine, 100 units/ml penicillin, 100 μg/ml streptomycin, and supplemented with 10% fetal bovine serum (Biological Industries Ltd., Cumbernauld, UK). The cells were maintained at 37 °C in a humidified incubator in an atmosphere of 5% CO_2_, and fresh growth medium was added to the cells every 3 to 4 days until the cells were ready for experimentation. The cells were incubated in serum-free medium for 48 h before use in all experiments, and all experiments were performed under serum-free conditions unless otherwise stated. All experiments were undertaken using cells at passage 6–10.

##### Cellular Treatments

Phenotypic differentiation of fibroblasts was induced by stimulation with 10 ng/ml TGF-β1 for 72 h according to time course and dose-response experiments and according to our previous protocols (33). Time course and dose-response experiments were also initially done to determine the optimum concentration and incubation times with BMP7. A dose of 400 ng/ml BMP7 was subsequently used in all experiments.

Two experimental cell systems were utilized to determine effects of BMP7 and related alterations in HA on TGF-β1 responsiveness, a prevention model and a reversal model of fibrosis. For the prevention model, cells were either treated with TGF-β1 and BMP7 at the same time (prevention I) or cells were pretreated with BMP7 prior to stimulation with TGF-β1 (prevention II). For the reversal model, cells were first stimulated with TGF-β1 to induce differentiation and then subsequently treated with BMP7. These treatment conditions and incubation times are more clearly demonstrated in Table 1.

**TABLE 1:** **Experimental cell treatment systems**

Treatment conditions	0–72 h	72–144 h
Untreated control		
TGF-β1	10 ng/ml TGF-β1	10 ng/ml TGF-β1
BMP7	400 ng/ml BMP7	400 ng/ml BMP7
Prevention I	10 ng/ml TGF-β1 and 400 ng/ml BMP7	10 ng/ml TGF-β1 and 400 ng/ml BMP7
Prevention II	400 ng/ml BMP7	10 ng/ml TGF-β1
Reversal	10 ng/ml TGF-β1	400 ng/ml BMP7

##### HA ELISA

An ELISA-like assay (HA ELISA) was commercially purchased (Corgenix, Broomfield, CO) and used to assess the concentrations of intracellular HA, HA in conditioned cell culture medium, and on the cell surface. Briefly, conditioned cell culture medium was removed and transferred into Eppendorf microcentrifuge tubes (soluble HA) and kept on ice. Cells were then incubated with trypsin/EDTA for 5 min at room temperature, transferred into microcentrifuge tubes, and centrifuged at 4000 × *g* for 5 min at 4 °C. The supernatant was transferred to Eppendorf microcentrifuge tubes, and trypsin was deactivated by heating to 90 °C for 5 min (cell-surface HA) and then kept on ice. The cell pellet was resuspended in dilute (10% v/v) RIPA buffer and kept on ice for 10 min. The solution was centrifuged again, and the supernatant transferred to fresh microcentrifuge tubes (intracellular HA). Samples were then analyzed by Corgenix HA ELISA kits (Corgenix), according to the manufacturer's protocol.

##### Reverse Transcription and Real Time Quantitative PCR (RT and RT-QPCR)

RT-QPCR was used to assess α-SMA (*ACTA2*), *CD44, Hyal1, Hyal2, HAS1, HAS2,* and *HAS3* mRNA expressions. Primers and probes for these genes were commercially designed and purchased from Applied Biosystems. The cells were grown in 35-mm dishes and washed with PBS before lysis with TRI Reagent and RNA purification according to the manufacturer's protocol. Reverse transcription was done using the high capacity cDNA reverse transcription kit according to the manufacturer's protocol (Applied Biosystems). This kit uses the random primer method for initiating cDNA synthesis. As a negative control, reverse transcription was done with sterile H_2_O replacing the RNA sample. QPCR was done using the ViiA7 real time QPCR system from Applied Biosystems in a final volume of 20 μl per sample as follows: 1 μl of reverse transcription product, 1 μl of target gene primers and probe, 10 μl of TaqMan Fast Universal PCR MasterMix, and 8 μl of sterile RNase-free water. Amplification was done using a cycle of 95 °C for 1 s and 60 °C for 20 s for 40 cycles. QPCR was simultaneously performed for ribosomal RNA (primers and probe commercially designed and purchased from Applied Biosystems) as a standard reference gene.

For assessment of CD44 variant isoform expression, custom primer pairs were designed and purchased from Invitrogen. Primer pairs used are shown in Table 2.

**TABLE 2:** **Forward and reverse primer pairs for CD44 standard and variant isoforms**

*CD44s*	Forward 5′-GCTACCAGAGACCAAGACACA-3′
	Reverse 5′-GCTCCACCTTCTTGACTCC-3′
*CD44v7*	Forward 5′-GAATCCCTGCTACCACAGCCTC-3′
	Reverse 5′-TCTCCCATCCTTCTTCCTGCTT-3′
*CD44v8*	Forward 5′-ATGTGTCTTGGTCTCGCGTT-3′
	Reverse 5′TCCCTGCTACCAATATGGACTC-3′
*CD44v3/7*	Forward 5′-TTATCTCCAGCACCACAGCCTC-3′
	Reverse 5′-TCTTGGTCTCCCATCCTTCTTC-3′
*CD44v7/8*	Forward 5′AGGAAGAAGGATGGATATGGACT-3′
	Reverse 5′-GTCTTGGTCTCGCGTTGTCA-3′

QPCR for custom primer pairs was done with the ViiA7 real time QPCR system from Applied Biosystems in a final volume of 20 μl per sample as follows: 1 μl of reverse transcription product; 0.6 μl of 10 μm target gene forward primer and 0.6 μl of 10 μm target gene reverse primer; 10 μl of Power SYBR Green Mastermix; and 7.8 μl of sterile RNase-free water. Amplification used a cycle of 95 °C for 15 s and 60 °C for 1 min for 40 cycles, followed by a melt-curve stage at 95 °C for 15 s, 60 °C for 1 min, and a final step of 95 °C for 15 s. QPCR was simultaneously performed for *GAPDH* mRNA expression (custom primers designed and purchased from Applied Biosystems) as a standard reference gene. As a negative control, PCR was performed with sterile H_2_O replacing the cDNA sample.

The comparative *CT* method was used for relative quantification of gene expression. The *CT* (threshold cycle where amplification is in the linear range of the amplification curve) for the standard reference gene (*rRNA/GAPDH*) was subtracted from the target gene *CT* to obtain the Δ*CT*. The mean Δ*CT* values for replicate samples were then calculated. The expression of the target gene in experimental samples relative to expression in control samples was then calculated using the following equation: 2^−(Δ^*^CT^*^(1) −^ ^Δ^*^CT^*^(2))^, where Δ*CT*(1) is the mean Δ*CT* calculated for the experimental samples, and Δ*CT*(2) is the mean Δ*CT* calculated for the control samples.

##### siRNA Transfection

Transient transfection of fibroblasts was done with specific siRNA nucleotides (Applied Biosystems) targeting *Hyal1, Hyal2,* or *CD44v7/8*. Transfection was done using Lipofectamine 2000 transfection reagent (Invitrogen) in accordance with the manufacturer's protocol. Briefly, cells were grown to 70% confluence in antibiotic-free medium in either 35-mm dishes or 8-well Permanox chamber slides. Transfection reagent (2% v/v) was diluted in Opti-MEM reduced growth medium (GIBCO) and left to incubate for 5 min at room temperature. The specific siRNA oligonucleotides were diluted in Opti-MEM reduced growth medium to achieve a final concentration of 30 nm. The transfection agent and siRNA mixtures were then combined and incubated at room temperature for an additional 20 min. The newly formed transfection complexes were subsequently added to the cells and incubated at 37 °C with 5% CO_2_ for 24 h in serum-free medium before experimentation. As a control, cells were transfected with negative control siRNA (a scrambled sequence that bears no homology to the human genome) (Applied Biosystems).

##### Immunocytochemistry

Cells were grown to 70% confluence in 8-well Permanox chamber slides. The culture medium was removed, and the cells washed with sterile PBS before fixation in 4% paraformaldehyde for 10 min at room temperature. After fixation, cells were permeabilized with 0.1% (v/v) Triton X-100 in PBS for 10 min at room temperature. Slides were blocked with 1% bovine serum albumin (BSA) for 1 h before a further washing step with 0.1% (w/v) BSA in PBS. Subsequently, the slides were incubated with the primary antibody diluted in 0.1% BSA and PBS for 2 h at room temperature. When visualizing HA, biotinylated HA-binding protein (bHABP) was used in place of primary antibody. After a further washing step, slides were incubated with AlexaFluor 488-conjugated and/or AlexaFluor 555-conjugated secondary antibodies for 1 h at room temperature. Avidin-FITC was used in place of a secondary antibody when visualizing HA. Cell nuclei were stained with Hoechst solution. Cells were then mounted and analyzed by confocal and fluorescent microscopy. A co-localization scatter plot was used to quantify the level of co-localization present between distinct antibody stains in selected experiments.

The following primary antibodies were used: mouse anti-human α-SMA (Sigma); rat anti-human CD44 antibody (Merck Millipore; A020); mouse anti-human EGFR antibody (Merck Millipore); biotinylated bHABP (Seikagaku Corp., Tokyo, Japan); mouse anti-human EEA1 antibody (Santa Cruz Biotechnology Inc., Heidelberg, Germany); mouse anti-human Cav1 antibody (Sigma); rabbit anti-human Hyal1 antibody and rabbit anti-human Hyal2 antibody (gift from Dr. Robert Stern, Department of Pathology, University of California, San Francisco); mouse anti-human CD44v7/8 antibody (Thermo Fisher Scientific, Leicestershire, UK); and cholera toxin subunit B Alexa Fluor 594 conjugate (Molecular Probes, Invitrogen). The following secondary antibodies were used: rabbit anti-rat AlexaFluor 555, goat anti-mouse AlexaFluor 488, goat anti-rabbit AlexaFluor 488, goat anti-rabbit AlexaFluor 555 (Merck Millipore); and avidin-FITC (Vector Laboratories, Burlingame, CA).

##### CD44 Variant Isoform Identification and Polymerase Chain Reaction (PCR)

Specific primer pairs were designed and purchased (Invitrogen) to target *CD44v7* and *CD44v8*. Two primer sets were used for *CD44v7* analysis as follows: set one forward 5′-TCAATGCTTCAGCTCCACCT-3′ and reverse 5′-TCTCCCATCCTTCTTCCTGCTT-3′; set two forward 5′-GAATCCCTGCTACCACAGCCTC-3′ and reverse 5′-CAAAGCCAAGGCCAAGAGGGATGC-3′. The following two primer sets were used for *CD44v8* analysis as follows: set one forward 5′-TCAATGCTTCAGCTCCACCT-3′ and reverse 5′-TCCCTGCTACCATATGGACTC-3′; set two forward 5′-ATGTGTCTTGGTCTCGCGTT-3′ and reverse 5′-CAAAGCCAAGGCCAAGAGGGATGC-3′.

Fragments were amplified using the Phusion High Fidelity DNA polymerase system (New England Biolabs, Inc.). Briefly, 4 μl of 5× High Fidelity buffer, 0.4 μl of dNTPs, 1 μl of 10 μm forward primer, 1 μl of 10 μm reverse primer, 0.2 μl of Phusion DNA polymerase, 10 ng of cDNA template, and distilled H_2_O were added to a final volume of 20 μl. Samples were PCR amplified with thermocycling conditions of: 98 °C for 30 s, 35 cycles of 98 °C for 10 s, 65 °C for 30 s, and 72 °C for 30 s. This was followed by a final extension at 72 °C for 10 min. The resultant PCR products were run on 1% agarose gels by flatbed electrophoresis. Bands were pictured and then excised for gel extraction and subsequent DNA sequencing (Eurofins MWG Operon, Ebersberg, Germany) to identify key CD44 variant isoforms amplified with BMP7 treatment.

##### Statistical Analysis

Two-tailed, unpaired Student's *t* tests were done to assess statistical differences between the two experimental groups. For experiments with multiple experimental conditions, one-way analysis of variance was used to identify statistical differences across groups, followed by post-test Bartlett's and two-tailed unpaired Student's *t* tests. Pearson's correlation was used to determine statistical significance of co-localization scatter plots. Graphical data are expressed as means ± S.E. All data were analyzed using GraphPad Prism version 4.01. *, *p* ≤ 0.05, and **, *p* ≤ 0.01, were considered statistically significant.

## RESULTS

### 

#### 

##### BMP7 Both Prevents and Reverses Differentiation of Fibroblasts to Myofibroblasts

**FIGURE 1.**
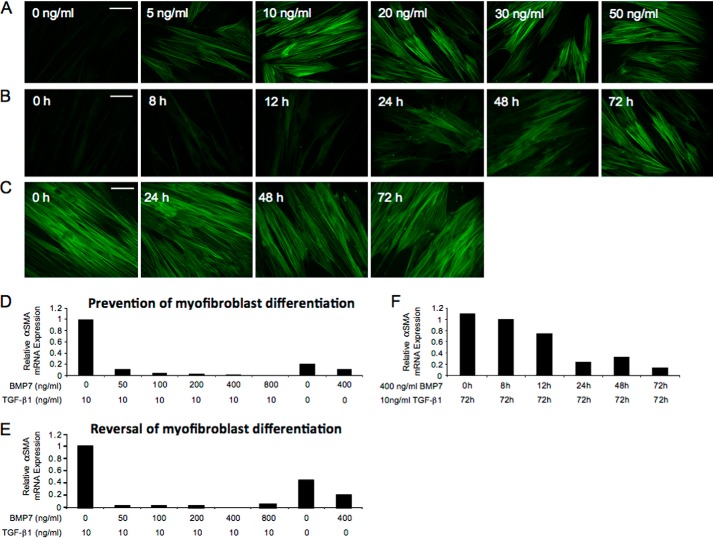
**10 ng/ml 72-h TGF-β1 induces stable myofibroblast differentiation, and prevention/reversal of myofibroblast phenotype is induced by 400 ng/ml 72-h BMP7.** *A–C,* fibroblasts were grown to ∼70% confluence before undergoing growth arrest in serum-free medium for 48 h. *A,* medium was replaced with serum-free medium containing 0, 5, 10, 20, 30, and 50 ng/ml TGF-β1 and the incubations continued for 72 h. *B,* medium was replaced with serum-free medium containing 10 ng/ml TGF-β1 for 0, 8, 12, 24, 48, and 72 h. *C,* medium was then replaced with serum-free medium containing 10 ng/ml TGF-β1 for 72 h and then subsequently again replaced with serum-free medium, and the incubations continued for a further 0, 24, 48, and 72 h. The cells were then fixed and stained for α-SMA and viewed under UV light. *D–F,* fibroblasts were grown to ∼70% confluence before undergoing growth arrest in serum-free medium for 48 h. *D,* cells were treated with 0, 50, 100, 200, 400, and 800 ng/ml BMP7 and the incubations continued for 72 h; then medium was replaced with serum-free medium containing 10 ng/ml TGF-β1 for 72 h. *E,* cells were treated with 10 ng/ml TGF-β1 for 72 h before undergoing treatment with 0, 50, 100, 200, 400, and 800 ng/ml BMP7 for a further 72 h. *F,* cells were treated with 400 ng/ml BMP7 for 0, 8, 12, 24, 48, and 72 h; then medium was replaced with serum-free medium containing 10 ng/ml TGF-β1 for a further 72 h. RNA extraction and RT-QPCR assessment for α-SMA mRNA expression was subsequently performed. Control experiments included cells treated with serum-free medium alone, cells treated with 10 ng/ml TGF-β1 alone, and cells treated with 400 ng/ml BMP7 alone. *White scale bars* represent 20 micrometers.

Exposure of human lung fibroblasts to different concentrations of TGF-β1 demonstrated that the minimum dose of TGF-β1 required to promote myofibroblast differentiation was 10 ng/ml. This was assessed by positive staining for the myofibroblast marker, α-SMA (Fig. 1*A*). Subsequent incubations of fibroblasts with 10 ng/ml TGF-β1 for different times demonstrated that 72-h incubations with this dose of TGF-β1 was required to promote complete myofibroblast differentiation (Fig. 1*B*). Subsequently, myofibroblast cultures were assessed to confirm stable differentiation of cells. Fibroblasts were initially stimulated with 10 ng/ml TGF-β1 to promote myofibroblast differentiation. Following this, TGF-β1 was removed from the cultures, and incubations were continued for a further 72 h. α-SMA staining persisted in the myofibroblast cultures following the removal of TGF-β1, indicating that these were stably differentiated cells, and their myofibroblast phenotype was not reversed by TGF-β1 removal (Fig. 1*C*).

Fibroblast and myofibroblast cultures were treated with different doses of BMP7 for various times to determine the effect on TGF-β1-driven myofibroblast differentiation. Initially, fibroblasts were pretreated with varying doses of BMP7 prior to 72 h of TGF-β1 exposure. These experiments demonstrated maximal prevention of TGF-β1-dependent myofibroblast differentiation by BMP7 at a dose of 400 ng/ml (Fig. 1*D*). Similarly, 400 ng/ml BMP7 was the maximal dose required to reverse TGF-β1-driven myofibroblast differentiation (Fig. 1*E*). Moreover, experiments were performed to determine the necessary BMP7 incubation times for maximal prevention/reversal of myofibroblast phenotype. Results demonstrated significant attenuation of α-*SMA* mRNA following 24 h of 400 ng/ml BMP7 exposure, with maximal attenuation demonstrated at 72 h (Fig. 1*F*).

**FIGURE 2.**
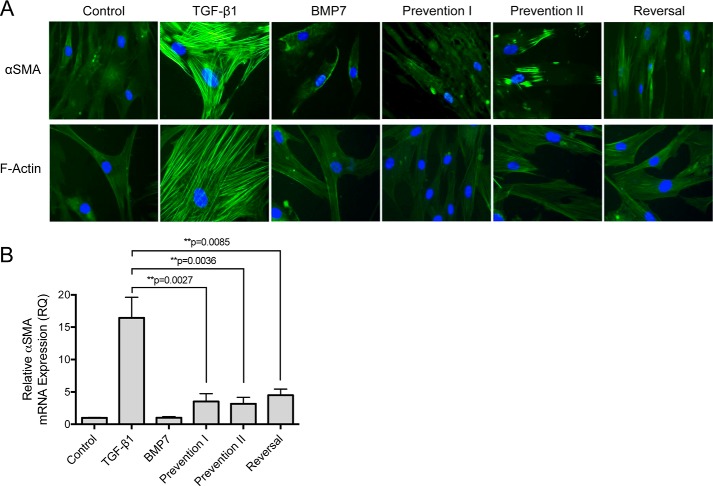
**BMP7 prevents and reverses myofibroblast phenotype.** *A,* fibroblasts were grown to ∼70% confluence before undergoing growth arrest in serum-free medium for 48 h. Cells were then treated under the various cytokines according to the experimental models described under “Experimental Procedures.” Subsequently, cells were fixed and stained for α-SMA (*top row*) and F-actin (*bottom row*). Images shown are representative of three individual experiments. *B,* fibroblasts were grown to confluence before growth arrest in serum-free medium for 48 h. Cells were then treated under the various experimental models as described above before extraction with TRIzol reagent. RNAs were purified, reverse-transcribed, and assessed for α-SMA mRNA expression using RT-QPCR. Results are shown as the mean ± S.E. of three individual experiments. Statistical analysis used the paired Student's *t* test with *p* < 0.05 considered as statistically significant.

All experiments were consequently performed at 72 h with 10 ng/ml TGF-β1 and at 72 h with 400 ng/ml BMP7. Confirmation experiments were repeated using these parameters. Stimulation of fibroblasts with 10 ng/ml TGF-β1 led to positive immunocytochemical staining for α-SMA. Unstimulated cells (control) and cells treated with BMP7 alone had minimal α-SMA staining as expected. Fibroblasts that were treated with BMP7 and TGF-β1 concurrently (prevention I) or fibroblasts that were first pretreated with BMP7 prior to incubation with TGF-β1 (prevention II) demonstrated minimal positive α-SMA staining. Similarly, fibroblasts that were first treated with TGF-β1 to induce myofibroblast differentiation and subsequently treated with BMP7 showed negligible staining for α-SMA (reversal) (Fig. 2*A*).

These results were further confirmed by assessing cytoskeletal reorganization in fibroblasts in response to BMP7. Cells were stained with FITC-phalloidin, a fluorescent probe that binds filamentous actin. Undifferentiated fibroblasts displayed a narrow spindle-shaped appearance with actin fibers forming a complex meshwork at the periphery of cells. No obvious bundles of actin fibers were evident. These are the results depicted in unstimulated cells and cells treated with BMP7 alone. Stimulation with TGF-β1 leading to myofibroblast differentiation led to prominent cytoskeletal reorganization with the actin filaments coalescing to form thick bundles that extend from end to end of the cells. However, pretreatment and co-treatment with BMP7 (prevention I and II), as well as treatment with BMP7 following TGF-β1 (reversal), prevented and reversed TGF-β1-driven reorganization of the filamentous actin cytoskeleton (Fig. 2*A*). Assessment of α-*SMA* expression in fibroblasts using quantitative PCR substantiated these results, demonstrating that BMP7 can both prevent and reverse TGF-β1-induced up-regulation in α-*SMA* mRNA expression (Fig. 2*B*).

##### BMP7 Drives Internalization of HA into Intracellular Vesicles in Fibroblasts and Myofibroblasts

**FIGURE 3.**
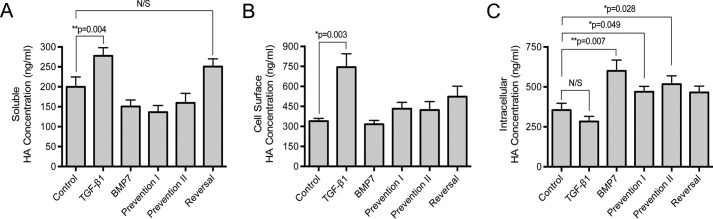
**BMP7 treatment alters cellular HA distribution resulting in internalization of HA in myofibroblasts.** Cells were grown to confluence in 35-mm dishes; growth-arrested for 48 h, and incubated with the indicated cytokine treatments in the experimental models as described under “Experimental Procedures.” HA ELISA was used to analyze the concentrations of HA in conditioned cell culture media (*A*), cell-surface trypsinates (*B*), and intracellular lysates (*C*). Results are expressed as the mean ± S.E. of three individual experiments. Statistical analysis used the paired Student's *t* test with *p* < 0.05 considered as statistically significant. *N/S*, not significant.

Previous studies have demonstrated an increase in extracellular and pericellular HA in response to TGF-β1 (33, 34). An ELISA-like assay was used to assess HA levels in the different BMP7 experimental models. The results demonstrated that TGF-β1 significantly increased HA levels above control in the extracellular and pericellular areas, although BMP7 did not (Fig. 3, *A* and *B*). In contrast, BMP7 treatments significantly increased levels of intracellular HA, although TGF-β1 did not (Fig. 3*C*).

**FIGURE 4.**
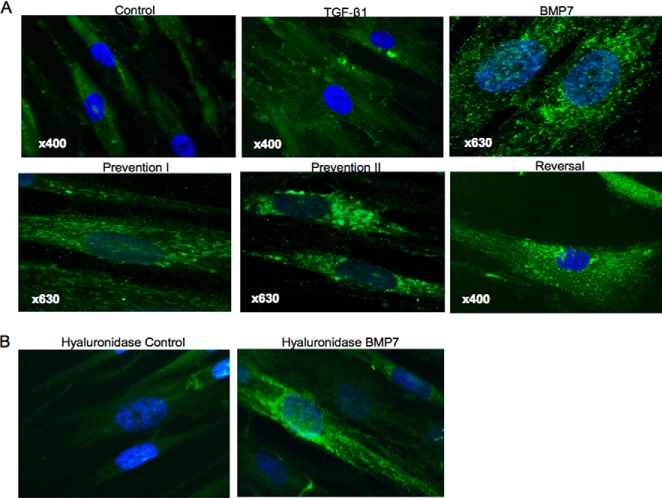
**BMP7 treatment internalizes HA into intracellular vesicles.** Cells were grown to 70% confluence before undergoing growth arrest in serum-free medium for 48 h. Cells were then treated under various cytokine conditions, according to the experimental models described under “Experimental Procedures” and subsequently fixed, permeabilized, and stained for HA using bHABP and avidin-FITC. *A*, HA visualization using laser scanning confocal microscopy following various cytokine treatment conditions. *B,* cells treated with hyaluronidase prior to fixation. All images are representative of three individual experiments. Original magnification is shown on individual panels.

HA localization and distribution in fibroblasts were assessed using immunocytochemical staining for FITC-labeled HABP. In unstimulated fibroblasts, intracellular HA was apparent and appeared to be distributed diffusely within the cytoplasm. In TGF-β1-induced myofibroblasts, HA was mainly localized at the cell surface. These findings are in keeping with our previous studies (12, 28). Fibroblasts treated with BMP7 demonstrated predominantly intracellular HA staining. However, the HA in these cells appeared to be localized in distinct punctate areas within the cytoplasm, in a pattern suggesting localization within vesicles. This pattern of HA staining was also demonstrated in fibroblasts pretreated and co-treated with BMP7 and TGF-β1 (Fig. 4*A*). To confirm that these areas of HA staining were localized within the cytoplasm rather than at the cell surface, fibroblasts were treated with *Streptomyces* hyaluronidase to remove any cell-surface HA prior to HA staining techniques. These experiments confirmed the intracellular localization of HA in response to BMP7 (Fig. 4*B*).

##### BMP7 Is Associated with Alterations in mRNA Expression of Hyaluronan Synthase 2 (HAS2), CD44, and Hyaluronidase 1 and 2

**FIGURE 5.**
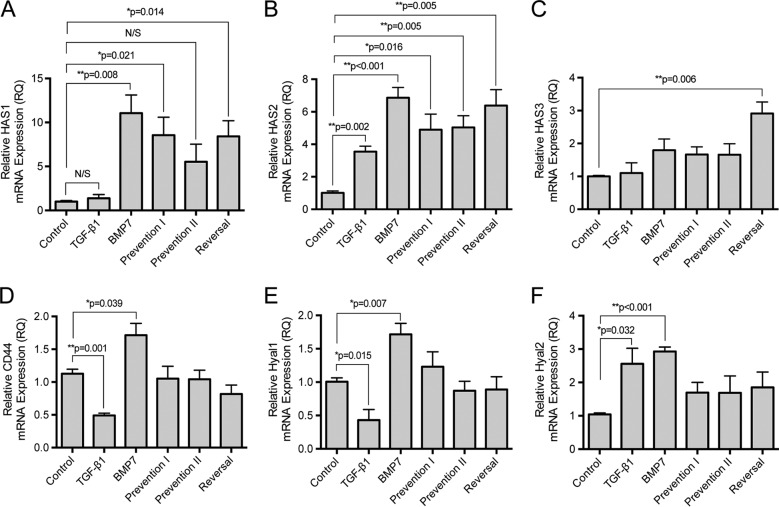
**BMP7 alters HA-associated gene expression.** Fibroblasts were grown to confluence before undergoing growth arrest in serum-free medium for 48 h. Cells were then treated under various cytokine conditions according to the experimental models described under “Experimental Procedures” before extraction with TRIzol reagent. RNAs were purified, reverse-transcribed and assessed for: HAS1 (*A*), HAS2 (*B*), HAS3 (*C*), CD44 (*D*), Hyal1 (*E*), and Hyal2 (*F*) mRNA expression using RT-QPCR. Results are shown as the mean ± S.E. of three individual experiments. Statistical analysis used the paired Student's *t* test with *p* < 0.05 considered as statistically significant, and comparison is made between control and all other treatment conditions.

Previous research in this area has demonstrated that TGF-β1 mediated fibroblast to myofibroblast differentiation, and generation of the pericellular HA coat is predominantly dependent on HA synthase isoforms HAS1 and HAS2 (26, 35). In these experiments TGF-β1 significantly increased *HAS1* and *HAS2* mRNA expression in the human lung fibroblasts (Fig. 5, *A* and *B*). In comparison, prevention and reversal of myofibroblast phenotype associated with BMP7 stimulation were associated with induction in gene expression of all three HA synthase isoforms (*HAS1, HAS2,* and *HAS3*) with a significant increase in *HAS2* over that induced by TGF-β1 (Fig. 5, *A–C*). Furthermore, although TGF-β1-driven fibroblast differentiation was associated with decreased *CD44* and *Hyal1* expression, BMP7 stimulation increased expression of both of these proteins (Fig. 5, *D* and *E*). *Hyal2* mRNA was increased, compared with controls, similarly by both TGF-β1 and BMP7 (Fig. 5*F*).

##### BMP7 Induces HA Internalization into Intracellular Catalytic Endosomes Containing Hyal1 and Hyal2

**FIGURE 6.**
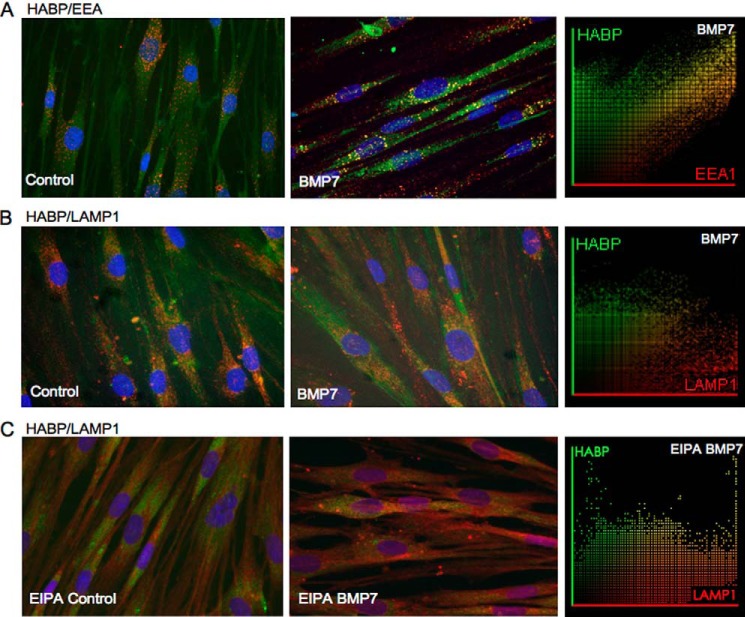
**Internalized HA is distributed into intracellular endosomes.** Fibroblasts were grown to 70% confluence before undergoing growth arrest in serum-free medium for 48 h. Cells were then incubated in serum-free medium alone or serum-free medium containing 400 ng/ml BMP7 and were subsequently fixed and stained for HABP (*green*)/EEA1 (*red*) (*A*), HABP (*green*)/LAMP1 (*red*) (*B*). Areas of co-localization appear as *yellow*, original magnification ×400. *C,* HABP (*green*)/LAMP1 (these cells having been additionally incubated with EIPA). Co-localization scatter plots were performed to confirm co-localization (depicted as *yellow areas*), and Pearson's correlation was used to confirm statistical significance. All images are representative of three individual experiments.

Co-staining of HA (HABP) with Cav1 (caveolin marker), EEA1 (endosomal marker), or LAMP1 (lysosomal marker) was used to assess the internalization of HA into caveolar, endosomal, or lysosomal vesicles, respectively. The presence of high cholesterol lipid rafts within the vesicular membrane was also investigated by co-staining for HA and the lipid raft marker (cholera toxin subunit B). Intracellular HA within the vesicles in BMP7-treated cells co-localized positively with the endosomal marker EEA1 (Fig. 6*A*) but not with LAMP1 (Fig. 6*B*). The level of association was confirmed by co-localization of scatter plots, which demonstrated significant areas of HA with EEA1 (Pearson's correlation coefficient mean *r* = 0.83 ± 0.072 (HA/EEA1) and mean *r* = 0.215 ± 0.053 (HA/LAMP1)). HA did not show co-localization with Cav1 or cholera toxin subunit B (data not shown). To confirm that the negative HABP and LAMP1 staining was not due to rapid HA degradation within lysosomes, cells were treated with EIPA (a known inhibitor of the Na^+^/H^+^ exchanger-1 known to activate hyaluronidase 2) before co-staining with HABP and LAMP1. The results demonstrated a slight but insignificant increase in co-localization between HABP and LAMP1 in EIPA- and BMP7-treated cells (Pearson's correlation coefficient mean *r* = 0.155 ± 0.074 for BMP7-treated cells *versus* mean *r* = 0.386 ± 0.170 for BMP7- and EIPA-treated cells) (Fig. 6*C*).

**FIGURE 7.**
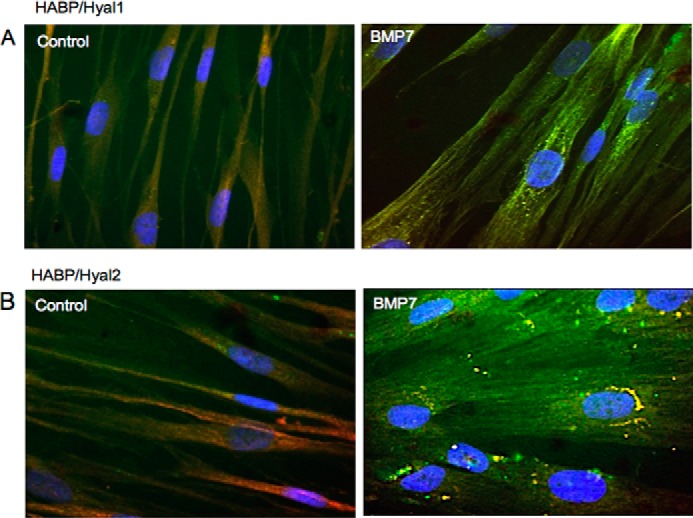
**Internalized HA is distributed into intracellular endosomes containing Hyal1 and Hyal2.** Fibroblasts were grown to 70% confluence before undergoing growth arrest in serum-free medium for 48 h. Cells were then incubated in serum-free medium alone or serum-free medium containing 400 ng/ml BMP7 and were subsequently fixed and stained for HABP (*green*)/Hyal1 (*red*) (*A*) and HABP (*green*)/Hyal2 (*red*) (*B*). Areas of co-localization appear as *yellow*, original magnification ×400.

**FIGURE 8.**
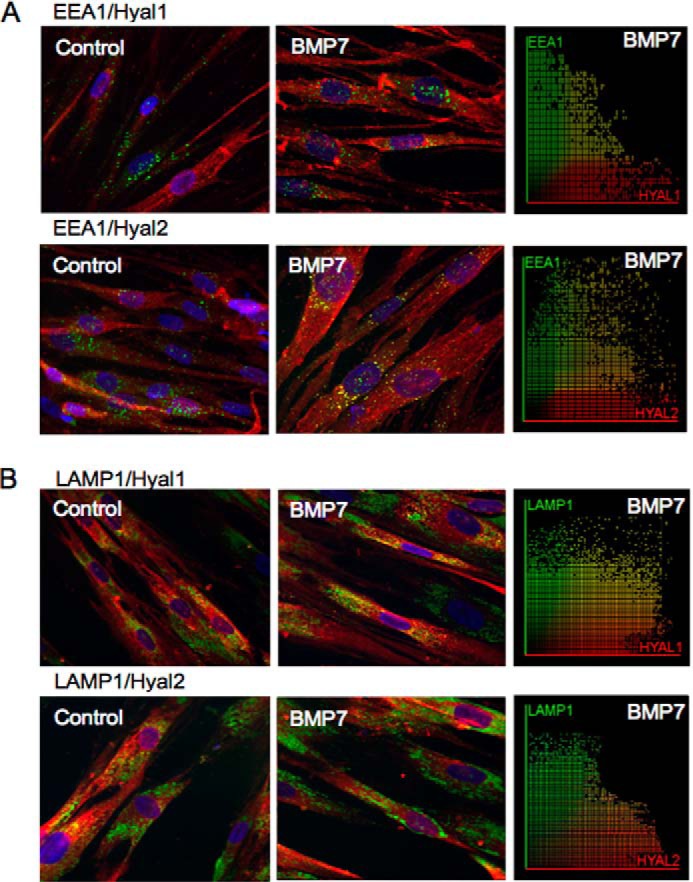
**Distribution of Hyal1 and Hyal2 in endosomes and lysosomes in fibroblasts.** Fibroblasts were grown to 70% confluence before undergoing growth arrest in serum-free medium for 48 h. Cells were then incubated in serum-free medium alone or serum-free medium containing 400 ng/ml BMP7 and were subsequently fixed and stained for EEA1 (*green*) and Hyal1 or Hyal2 (*red*) (*A*) and LAMP1 (*green*) and Hyal1 or Hyal2 (*red*) (*B*). Areas of co-localization appear as *yellow*, original magnification ×400. Co-localization scatter plots were performed to confirm co-localization (depicted as *yellow areas*), and Pearson's correlation was used to confirm statistical significance.

Enzymatic degradation of HA is initiated by the Hyal group of enzymes. In somatic tissues, Hyal1 and Hyal2 function as the major hyaluronidases for HA degradation. Hyal1 is found predominantly intracellularly, and Hyal2 can be found both intracellularly and at the cell surface (36). To determine whether HA internalization by BMP7 stimulation may be associated with its breakdown, immunocytochemistry was used to co-stain intracellular HA (*i.e.* after hyaluronidase treatment of intact cells) with Hyal1 and Hyal2. Both Hyal1 and Hyal2 demonstrated some co-localization with intracellular HA. However, the distribution of HA-Hyal1 and HA-Hyal2 endosomes within the cells varied. HA-Hyal1 endosomes were distributed in the perinuclear region (Fig. 7*A*), whereas the HA-Hyal2 endosomes were located at the nuclear margin (Fig. 7*B*). Subsequent staining for Hyal1 and Hyal2 with EEA1 and LAMP1 was performed. The results indicated that both endosomes and lysosomes contained Hyal1 and Hyal2, with endosomes containing relatively more Hyal2 than Hyal1 and lysosomes containing more Hyal1 than Hyal2 (Pearson's correlation coefficient mean *r* = 0.447 ± 0.048 Hyal1/EEA1, mean *r* = 0.625 ± 0.220 Hyal2/EEA1, mean *r* = 0.748 ± 0.146 Hyal1/LAMP1, and mean *r* = 0.131 ± 0.052 Hyal2/LAMP1) (Fig. 8, *A* and *B*).

##### BMP7 Drives Marked Alterations in CD44 Distribution and CD44 Variant Isoform Expression in Fibroblasts

**FIGURE 9.**
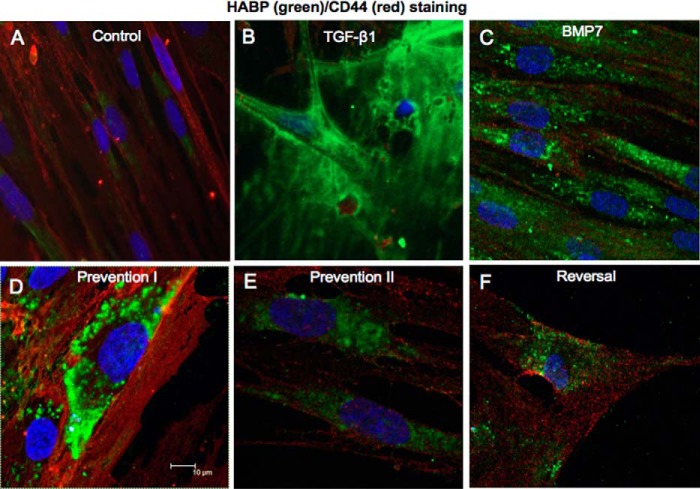
**BMP7 redistributes HA and CD44 in myofibroblasts.** Fibroblasts were grown to 70% confluence before undergoing growth arrest in serum-free medium for 48 h. Cells were then incubated under the various cytokine treatments according to the experimental models described under “Experimental Procedures.” Cells were subsequently fixed and stained for HABP (*green*) and CD44 (*red*). Areas of co-localization appear as *yellow*, original magnification ×400. All images are representative of three individual experiments.

**FIGURE 10.**
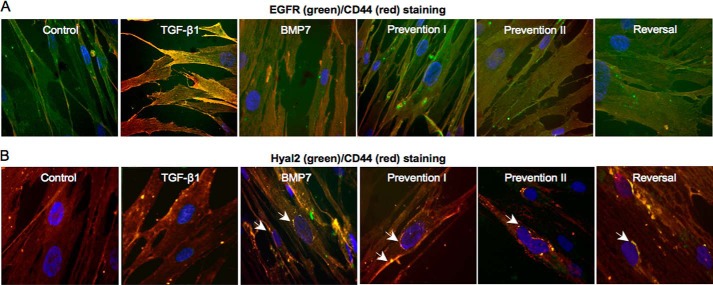
**BMP7 redistributes CD44, EGFR, and Hyal2 in myofibroblasts.** Fibroblasts were grown to 70% confluence before undergoing growth arrest in serum-free medium for 48 h. Cells were then incubated under the various cytokine treatments according to the experimental models described under “Experimental Procedures.” Cells were subsequently fixed and stained for EGFR (*green*) and CD44 (*red*) (*A*) and Hyal2 (*green*) and CD44 (*red*) (*B*). Areas of co-localization appear as *yellow*, original magnification ×400. All images are representative of three individual experiments.

CD44 is the principal cell-surface HA receptor. This trans-membrane glycoprotein can exist as multiple isoforms due to variable splicing of exons and exhibits considerable protein diversity (37). Furthermore, its coupling and association with other proteins and its distribution within the cell membrane are markedly influenced by distinct growth factors (12, 28, 38). Our previous work has outlined the importance of TGF-β1-driven CD44 redistribution and CD44/EGFR coupling within the cell membrane in mediating fibroblast to myofibroblast differentiation (12, 27). Although TGF-β1 attenuated total *CD44* mRNA expression, BMP7 increased *CD44* mRNA (Fig. 5*D*). We subsequently used immunocytochemistry to assess the influence of BMP7 on protein expression and distribution of CD44 in relation to HA. In keeping with our previous published research, CD44 was distributed in distinct areas mainly at the cell surface in resting fibroblasts (Fig. 9*A*). In TGF-β1-induced myofibroblasts, CD44 had lower levels of expression and was distributed diffusely at the cell surface. Increased pericellular HA was prominent in these cells (Fig. 9*B*). BMP7 stimulation alone, or in conjunction with TGF-β1 according to our prevention/reversal experimental models, led to cell surface as well as intracellular staining of CD44 (Fig. 9, *C–F*). CD44 appeared to be localized within distinct areas both at the cell membrane and in the cytoplasm in these cells. However, CD44 did not co-localize with HA within the majority of intracellular vesicles. Co-staining for CD44 with EGFR demonstrated dissociation between CD44 and EGFR in fibroblasts co-treated with BMP7 and TGF-β1, depicted as reduced yellow staining compared with cells treated with TGF-β1 alone (Fig. 10*A*). However, co-staining for CD44 and Hyal2 demonstrated association between these proteins particularly at the cell surface and the nuclear margin in response to BMP7 (Fig. 10*B, arrows*). No similar relationship could be identified between CD44 and Hyal1 (data not shown).

##### CD44v7/8 Is Key in Mediating BMP7-driven Prevention and Reversal of Myofibroblast Phenotype

**FIGURE 11.**
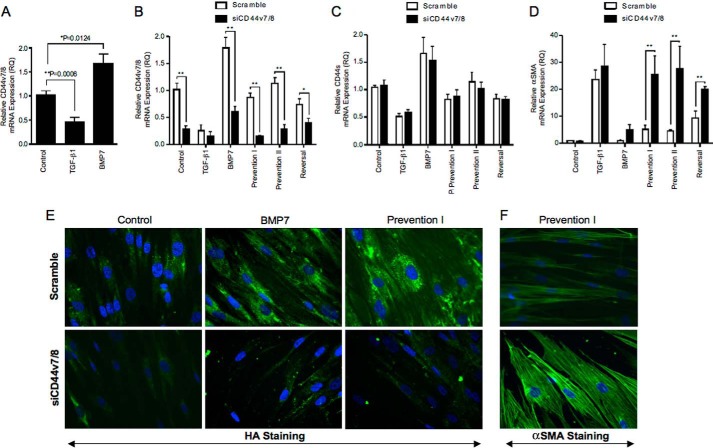
**BMP7 drives changes in variant CD44 gene expression, which are essential for mediating BMP7-driven alterations in myofibroblast phenotype.** *A,* fibroblasts were grown to confluence before growth arrest in serum-free medium for 48 h. Cells were then treated under various cytokine conditions, as indicated, before extraction with TRIzol reagent. RNAs were purified, reverse-transcribed, and assessed for CD44v7/8 mRNA expression using RT-QPCR. *B–D,* cells were grown to subconfluence and transfected with specific siRNA nucleotides targeting CD44v7/8 for 24 h prior to the indicated cellular treatments. Cells were then treated under various cytokine conditions according to experimental models as described under “Experimental Procedures” before extraction with TRIzol reagent. RT-QPCR was used to assess CD44v7/8 (*B*), CD44s (*C*), and α-SMA (*D*) mRNA expression. Results are shown as the mean ± S.E. of three individual experiments. Statistical significance used the paired Student's *t* test, and *p* < 0.05 was considered as significant. *E* and *F,* cells were grown to subconfluence and transfected with specific siRNA nucleotides targeting CD44v7/8 24 h prior to the indicated cellular treatments. Cells were subsequently fixed and stained for HABP (*E*) and α-SMA (*F*) as shown. Original magnification ×400. Images shown are representative of three individual experiments.

The type of CD44 variant isoform expressed can relate to different and sometimes opposing cellular functions (39–41). The influence of TGF-β1 and BMP7 on CD44 variant isoforms in our cell systems was therefore investigated. Primers for *CD44* variant isoforms were designed to amplify CD44 exon targets between specific primers. These primer pairs and conventional RT-PCR expression of individual *CD44* isoforms were used to assess the response to TGF-β1 and BMP7 (data not shown). *CD44v7/8* was shown to have prominent expression in BMP7-treated cells. The PCR products were subsequently excised and sequenced to confirm expression. Specific primers were designed for QPCR amplification with results demonstrating down-regulation of all variant isoform expression with TGF-β1 (data not shown). In comparison, significant up-regulation of variant isoform *CD44v7/8* was demonstrated with BMP7, although significant down-regulation was observed with TGF-β1, indicating a possible role for this isoform in BMP7 prevention of TGF-β1 myofibroblast differentiation (Fig. 11*A*). *CD44v7/8* had moderate level expression in control fibroblasts, with threshold cycles of amplification ranging between 28 and 32 out of 40. The threshold cycle varied between 27 and 29 for BMP7 treatments and was higher (threshold cycles 32–35) and therefore demonstrated low level expression in fibroblasts stimulated with TGF-β1.

siRNAs targeting *CD44v7/8* were used to determine the role of this variant isoform in the BMP7-mediated changes in HA internalization and inhibition of myofibroblast phenotypes. Successful knockdown of *CD44v7/8* expression following siRNA treatment compared with scrambled controls was initially confirmed (*white bars* compared with *black bars*, Fig. 11*B*). To confirm specificity of the siRNA for this variant of *CD44*, *CD44v7/8* siRNA transfected fibroblasts were evaluated for expression of *CD44s* and total *CD44*. Neither *CD44s* expression (Fig. 11*C*) nor total *CD44* expression (data not shown) demonstrated significant changes in expression following transfection with *CD44v7/8* siRNA compared with scrambled controls. The influence of knocking down *CD44v7/8* on α-*SMA* mRNA expression was subsequently assessed (Fig. 11*D*). Significant increases in α-*SMA* mRNA expression were observed in BMP7-treated cells incubated with the *CD44v7/8* siRNA compared with scrambled siRNA, although no difference was observed in the TGF-β1-treated cells. This suggests that knockdown of this variant isoform decreased the ability of BMP7 to prevent and reverse the TGF-β1-mediated myofibroblast phenotype. Immunostaining of cells for α-SMA also confirmed maintenance of the myofibroblast phenotype despite BMP7 treatment, with more prominent α-SMA staining present in cells that were incubated with the siRNA targeting *CD44v7/8* (Fig. 11*F*). Moreover, knockdown of *CD44v7/8* also inhibited BMP7-mediated HA internalization into intracellular endosomes, with *CD44v7/8* siRNA-treated cells showing minimal HA internalization (Fig. 11*E*).

##### Cell-surface Hyal2 and the NHE1 Are Also Essential for Mediating BMP7-driven Prevention and Reversal of Myofibroblast Phenotype

**FIGURE 12.**
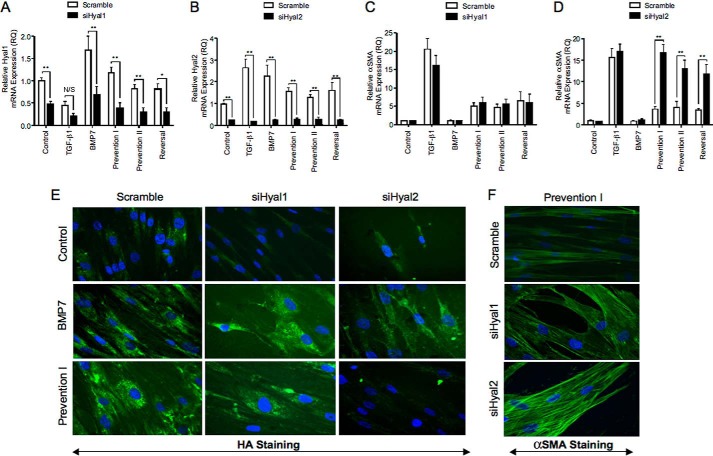
**BMP7-driven alterations in myofibroblast phenotype are Hyal2-dependent.** Cells were grown to subconfluence and transfected with specific siRNA nucleotides targeting Hyal1 (*A* and *C*) and Hyal2 (*B* and *D*) for 24 h prior to the indicated cellular treatments according to experimental models before extraction with TRIzol reagent. RT-QPCR was used to assess Hyal1 (*A*), Hyal2 (*B*), and α-SMA (*C* and *D*) mRNA expression. Results are shown as the mean ± S.E. of three individual experiments. Statistical significance used the paired Student's *t* test, and *p* < 0.05 was considered as significant. *E* and *F,* cells were grown to subconfluence before transfection with specific siRNA nucleotides targeting Hyal1 or Hyal2 for 24 h prior to the indicated cellular treatments. Cells were subsequently fixed and stained for α-SMA (*E*) and HABP (*f*). Original magnification ×400 Images shown are representative of three individual experiments.

To determine the role of the hyaluronidases (Hyal1 and Hyal2) in mediating the BMP7-driven changes in prevention and reversal of myofibroblast phenotypes, siRNAs targeting *Hyal1* and *Hyal2* were used to knock down expression of these proteins. Initial experiments to determine knockdown were initially performed (Fig. 12, *A* and *B*). The effect of inhibiting *Hyal1* and *Hyal2* on α-*SMA* mRNA expression in myofibroblasts was then assessed. Knockdown of *Hyal1* siRNA did not influence α-*SMA* expression in our experimental systems (Fig. 12*C*). In contrast, Fig. 12*D* showed that *Hyal2* siRNA effectively inhibited BMP7-dependent attenuation of α-*SMA* mRNA expression in all treatments. This indicated that knockdown of *Hyal2* inhibited the ability of BMP7 to prevent/reverse TGF-β1-induced myofibroblast phenotype. Immunocytochemical staining for α-SMA protein following treatment with *Hyal1* and *Hyal2* siRNA demonstrated similar results, with the presence of increased stress fiber formation and α-SMA staining following knockdown of *Hyal2* but not knockdown of *Hyal1* (Fig. 12*F*). HA staining also demonstrated that inhibiting *Hyal2* but not *Hyal1* attenuated BMP7-mediated HA internalization into cytosolic endosomes (Fig. 12*E*).

**FIGURE 13.**
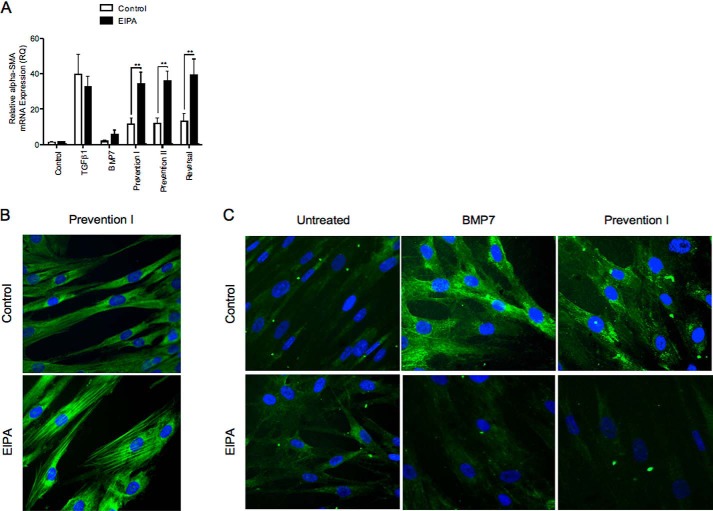
**Blockade of NHE1 with EIPA inhibits BMP7-driven prevention and reversal of myofibroblast phenotype.** *A,* cells were grown to confluence, growth-arrested, and then treated under various cytokine conditions as indicated, before extraction with TRIzol reagent. RNAs were purified, reverse-transcribed, and assessed for α-SMA mRNA expression using RT-QPCR. Results are shown as the mean ± S.E. of three individual experiments. Statistical significance was assessed using the paired Student's *t* test, and *p* < 0.05 was considered as significant. *B* and *C,* fibroblasts were grown to 70% confluence before growth arrest in serum-free medium for 48 h. Cells were then incubated under the various cytokine treatments, as indicated, and subsequently fixed and stained for α-SMA (*B*) and HABP (*C*). Original magnification ×400, and all images are representative of three individual experiments.

**FIGURE 14.**
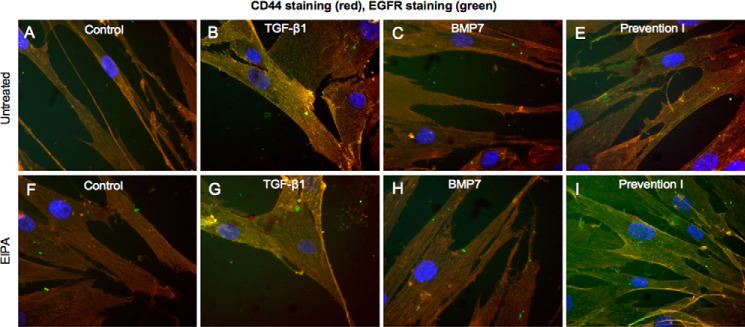
**Blocking NHE1 with EIPA prevents BMP7-driven EGFR/CD44 disassociation.** Fibroblasts were grown to 70% confluence before growth arrest in serum-free medium for 48 h. Cells were then incubated under the various cytokine treatments according to experimental models as described under “Experimental Procedures.” Cells were subsequently fixed and stained for EGFR (*green*) and CD44 (*red*). Areas of co-localization appear as *yellow*, original magnification ×400. All images are representative of three individual experiments.

**FIGURE 15.**
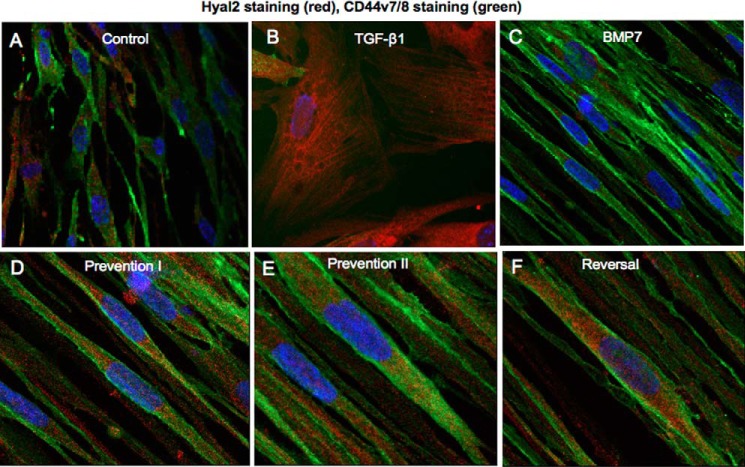
**CD44v7/8 and Hyal2 do not associate at the cell surface.** Fibroblasts were grown to 70% confluence before growth arrest in serum-free medium for 48 h. Cells were then incubated under the various cytokine treatments according to experimental models as described under “Experimental Procedures.” Cells were subsequently fixed and stained for Hyal2 (*red*) and CD44v7/8 (*green*). Areas of co-localization appear as *yellow*, original magnification ×400. All images are representative of three individual experiments.

Hyal2 possesses an acidic pH optimum (pH 3.7). Hyal2 has previously been demonstrated to be present in the cytoplasm contained within lysosomes (42). However it is also present at the cell surface linked to the plasma membrane via a glycosylphosphatidylinositol anchor (43). Hyal2 at the cell surface is activated to its pH optimum through the creation of an acidic microenvironment by NHE1 (44). NHE1 and Hyal2 have also been shown to form a complex with CD44s to promote breast tumor cell invasiveness (44). EIPA is a known inhibitor of NHE1. To determine the role of cell-surface Hyal2 *versus* intracellular Hyal2 in BMP7-mediated prevention/reversal of TGF-β1-induced myofibroblast phenotype, NHE1 action was blocked with EIPA to inhibit cell-surface Hyal2 activity. Myofibroblasts exposed to this treatment did not demonstrate prevention and reversal of phenotype by BMP7 as assessed by α-*SMA* mRNA expression (Fig. 13*A*). They also demonstrated increased α-SMA by immunocytochemistry (Fig. 13*B*) and reduced HA internalization in response to BMP7 (Fig. 13*C*). Furthermore, NHE1 inhibition also appeared to lead to re-association between CD44 and EGFR membrane proteins despite co-treatment of the cells with BMP7 and TGF-β1 (Fig. 14). We also investigated a link between variant CD44v7/8 and Hyal2 (Fig. 15). Dual staining for CD44v7/8 (Fig. 15, *green*) and Hyal2 (*red*) was performed. The results showed that fibroblasts demonstrated constitutive protein expression of CD44v7/8 at the cell surface under basal conditions. Hyal2 and CD44v7/8 did not co-localize under basal conditions (Fig. 15*A*). In TGF-β1-induced myofibroblasts, CD44v/8 (*green*) staining was attenuated in line with its reduced mRNA expression (Fig. 15*B*). In BMP7-treated cells (Fig. 15, *C–F*), CD44v7/8 demonstrated significantly increased expression at the cell surface; however, no evidence of association was found between Hyal2 and CD44v7/8 under any of the BMP7 treatment conditions.

## DISCUSSION

Aberrant expression of the cytokine TGF-β1 has been widely implicated in progressive fibrosis (45–47). Alongside its direct effects on extracellular matrix turnover, TGF-β1 is the principal factor known to drive myofibroblast formation, and it is the persistence of these contractile cells within tissues that predicts progression of fibrotic disease (48–53). Our previous work has shown that HA is a major contributor to TGF-β1-driven fibrosis. TGF-β1 promotes HAS2-driven HA generation alongside increased transcription and translation of the HA-binding protein tumor necrosis factor-stimulating gene-6 (TSG-6) (12, 33). TSG-6 binds and stabilizes the newly generated HA matrix forming TSG-6-dependent HA pericellular coats around individual myofibroblasts (54). These HA coats are essential for driving CD44 redistribution into lipid raft-rich areas within the cell membrane where it interacts with the EGF receptor, which triggers a cascade of downstream intracellular signaling events essential for fibroblast to myofibroblast differentiation (27). Thus, the HA pericellular coat is essential in maintaining myofibroblast phenotype and in promoting the pro-fibrotic actions of TGF-β1. Alternative forms of HA matrix have also been identified in fibroblasts and have distinct functions. Our group has previously shown that the formation of a spiculated HA pericellular matrix associated with IL-1β stimulation was not associated with myofibroblast formation. Instead, it led to enhanced CD44 expression and its co-localization with the adhesion molecule ICAM-1 resulting in fibroblast-monocyte binding (28). Thus, the ability of the HA matrix to regulate cellular functions is well recognized. Therefore, insight into how BMP7 may orchestrate an anti-fibrotic HA matrix that antagonizes the pro-fibrotic TGF-β1-driven matrix becomes imperative for understanding the mechanisms through which BMP7 can prevent and reverse fibrosis. However, in this work we demonstrate that BMP7-driven prevention and reversal of TGF-β1-mediated myofibroblast formation was not associated with a distinct pericellular HA matrix. Rather, BMP7 promoted internalization of HA and loss of the TGF-β1-driven HA pericellular matrix. HA was internalized into intracellular catalytic endosomes containing Hyal1 and Hyal2. Furthermore, internalization of HA through this pathway was dependent on dissociation between CD44 and EGFR (55), and it was also dependent on cell-surface Hyal2 and recruitment of the CD44 variant isoform, CD44v7/8.

The principal HA receptor, CD44, is widely expressed in mammalian tissues and has been shown to have important roles in inflammation, tumor progression, embryogenesis, and immune cell activation as well as fibrosis (16, 37, 56–58). This cell adhesion molecule can function as a co-receptor as well as bind growth factors and other (non-HA) components of the extracellular matrix (37). It contains a short cytoplasmic tail with multiple phosphorylation sites, a transmembrane domain, and an extracellular domain that contains an amino-terminal HA binding domain containing a link module (55, 59, 60). Alternative splicing of exons encoding the extracellular domain leads to formation of variant CD44 isoforms (CD44v) and is the basis for structural and functional diversity of this protein. Standard CD44 (CD44s) is the most abundantly expressed and simplest of all CD44 variants, containing only the common extracellular amino-terminal domain, transmembrane domain, and cytoplasmic region. All other variants have this overall pattern but include additional amino acids in the stem structure to produce different sized molecules. Other researchers, particularly in the cancer field, have demonstrated that the CD44 variant isoforms can have distinct modes of action and divergent functions (61–64). In the field of renal disease, the overexpression of the variant isoform CD44v3 has been shown to limit the actions of TGF-β1 and confer renoprotective properties in mice in a model of unilateral ureteric obstruction (39). In pulmonary fibrosis, variant isoforms CD44v6 and CD44v9 have shown reduced expression compared with normal lung tissues (65). Other than these two studies, the potential role of variant CD44 isoforms in the promotion and/or prevention of fibrotic disease has received little attention. In our previous work, we have demonstrated the importance of the pericellular HA matrix in dictating overall CD44 membrane interactions and membrane distribution (54). In this study we have shown that TGF-β1 and BMP7 differentially regulate overall CD44 expression. Although TGF-β1 attenuates CD44 expression, BMP7 augments CD44 expression, in particular significantly increasing expression of the variant isoform CD44v7/8. This CD44 variant is translated from mRNA containing exons 11 and 12, in addition to the standard exons encoding CD44s (exons 1–5 and 15–19). Inhibiting this CD44 variant blocks BMP7-mediated HA internalization as well as inhibiting the actions of BMP7 in prevention and reversal of the TGF-β1-induced myofibroblast phenotype. Thus, it is clear that CD44v7/8 is essential in mediating the beneficial actions of BMP7 in human fibroblasts.

The mechanisms through which this occurs are not yet clearly identified and will be the subject of future study. However, a number of potential mechanisms may be involved. Previous research has shown that in addition to its role as a signaling receptor, CD44 also has an important role in internalization of HA for degradation (66). Hence, this variant isoform may specialize in HA internalization rather than the maintenance of a TSG-6-HA-CD44-dependent pericellular coat. Likewise, other studies have shown that the affinity of the HA-CD44 bond can vary, and the strength of the HA-CD44 interaction can influence the resulting CD44-driven cellular responses (42, 67–69). CD44v7/8 may exhibit either enhanced or dampened affinity for HA, which promotes dissolution of the HA coat and interferes with TGF-β1-dependent signal transduction. Alternatively, in preference to interaction with the EGF receptor, CD44v7/8 may interact with other, as yet unidentified, membrane components that facilitate BMP7-driven responses. Thus, studies of the CD44 variant isoform (particularly CD44v7/8) mechanism of alternative splicing, modes of action, and distinct functions are potentially a promising field in fibrosis research.

The data presented in this study also denote the importance of Hyal2 in mediating the actions of BMP7. Hyal2 is an acid active enzyme generally found in two forms. It has been reported to exist in the cytoplasm within lysosomes (42). It has also been found anchored to the cell surface via a glycosylphosphatidylinositol link (43). Cell-surface Hyal2 is thought to have weaker enzymatic activity, but it has been reported to have a number of nonenzymatic functions, including forming complexes with other membrane proteins and regulation of Smad-driven promoters (70). The bulk of HA cleavage by cell-surface Hyal2 is thought to occur in acidic pockets generated by NHE1, which catalyzes the extrusion of intracellular H^+^ in exchange for extracellular Na^+^ (44). In our experiments, blocking NHE1 activity and inhibiting Hyal2 had the same effects, which led us to propose that cell-surface Hyal2 and not intracellular Hyal2 is central to mediating BMP7 actions. Activated Hyal2 catalyzes high molecular mass HA into intermediate size fragments of ∼20 kDa (50 disaccharides). Thus, Hyal2 in our system may also be contributing to dissolution of the HA coat with subsequent CD44v7/8-mediated internalization of the HA fragments. In breast cancer cells, cell-surface Hyal2 has been shown to form a functional complex with CD44 and NHE1. This led us to assume that a similar process may be occurring in our cell systems utilizing the variant isoform CD44v7/8 along with NHE1 and Hyal2. However, no co-localization was apparent between this variant isoform and Hyal2 in our experiments. In other work, overexpression of Hyal2 led to CD44 shedding from lipid rafts (71). Thus, the functional link between CD44v7/8 and Hyal2 in promoting BMP7 actions in our cell system is not yet understood. However, we have also shown that NHE1 and cell-surface Hyal2 play significant roles in maintaining dissociation of the CD44-EGFR complex by BMP7. We know from our previous work that the CD44-EGFR complex promoted by TGF-β1 resides within lipid rafts at the cell membrane. Therefore, Hyal2 (activated by NHE1) may be necessary to shed CD44 from lipid raft membrane fractions following BMP7 stimulation, resulting in its dissociation from EGFR.

HA internalized as a consequence of BMP7 action was found contained within intracellular endosomes. The HA-containing endosomes also positively stained for Hyal1 and to a greater extent, Hyal2. The HA-Hyal1- and HA-Hyal2-containing endosomes appeared to occupy distinct areas within the cytoplasm, with the HA-Hyal1 endosomes being contained within the perinuclear region and the HA-Hyal2 endosomes being located at the nuclear margin. These findings led us to propose that HA internalized by BMP7 was targeted for degradation. The different cytoplasmic localization of HA-Hyal1- and HA-Hyal2-containing endosomes supports a stepwise process of HA degradation similar to that reported by previous investigators (72, 73). It was surprising that internalized HA did not associate significantly with lysosomes. It is possible that the little association between HA and LAMP1 could be due to rapid HA degradation within lysosomes, thus rendering the HA undetectable. Inhibition of Hyal2 activity using EIPA did not significantly increase the association between HA and LAMP1. However, EIPA does not inhibit Hyal1 activity, and it may not markedly influence Hyal2 activity in already acidic lysosomes. Thus, these experiments do not preclude the internalization of HA into lysosomes as well as endosomes for degradation.

The observation of intracellular HA in this study adds to a growing number of recent studies demonstrating intracellular HA in various states of synthesis or degradation. Increasingly, data in this field suggest that intracellular HA may also be involved in regulating cellular function. HA within cells has been localized to diverse locations, including nuclei, nucleoli, cytoskeleton, and endoplasmic reticulum, and it has been implicated in processes such as cell motility, cell cycle control, RNA splicing, and regulation of the mitotic spindle (74–83). Furthermore, the presence of intracellular hyaladherins (such as CD44 and RHAMM) and the indication that HA may be synthesized intracellularly raise the question as to whether intracellular HA and its associates can serve as-yet undefined functions (84). In mesangial and other cells, intracellular accumulation of HA has been associated with a high glucose environment and has been linked to the process of cell autophagy (85). In our cell systems, we showed that although BMP7 drove increased CD44v7/8 expression at the cell surface, leading to internalization of other CD44 molecules into the cytoplasm and subsequent co-localization with HA-Hyal2 vesicles at the nuclear margin. The functional consequences of these changes are unclear from this study, However, it has been shown in previous research that the intracellular domain of CD44 can undergo cleavage and translocate to the nucleus resulting in altered gene transcription (86). Hence the study of intracellular HA and its associates may be another important avenue to pursue in understanding the anti-fibrotic actions of BMP7.

In summary, this study has shown that BMP7 antagonizes the pro-fibrotic effects of TGF-β1 in fibroblasts and myofibroblasts through dissolution and internalization of the TSG-6 and CD44-EGFR-dependent pericellular HA coat into hyaluronidase-containing endosomes. These events are dependent on increased expression of CD44v7/8 at the cell surface, along with the combined actions of cell-surface Hyal2 and NHE1, thus identifying key mediators of BMP7 action important for future study.

## Footnotes

2 The abbreviations used are:

HA: hyaluronan
QPCR: quantitative PCR
bHAPB: biotinylated HA-binding protein
EIPA: 5-(*N*-ethyl-*N*-isopropyl) amiloride
EGFR: EGF receptor
Hyal1: hyaluronidase-1
Hyal2: hyaluronidase-2
NHE1: Na/H exchanger
αSMA: α-smooth muscle actin.
